# Pre-Treatment with Amifostine Protects against Cyclophosphamide-Induced Disruption of Taste in Mice

**DOI:** 10.1371/journal.pone.0061607

**Published:** 2013-04-23

**Authors:** Nabanita Mukherjee, Brittany L. Carroll, Jeffrey L. Spees, Eugene R. Delay

**Affiliations:** 1 Department of Biology, University of Vermont, Burlington, Vermont, United States of America; 2 Department of Medicine, Stem Cell Core, Colchester, Vermont, United States of America; Barnard College, Columbia University, United States of America

## Abstract

Cyclophosphamide (CYP), a commonly prescribed chemotherapy drug, has multiple adverse side effects including alteration of taste. The effects on taste are a cause of concern for patients as changes in taste are often associated with loss of appetite, malnutrition, poor recovery and reduced quality of life. Amifostine is a cytoprotective agent that was previously shown to be effective in preventing chemotherapy-induced mucositis and nephrotoxicity. Here we determined its ability to protect against chemotherapy-induced damage to taste buds using a mouse model of CYP injury. We conducted detection threshold tests to measure changes in sucrose taste sensitivity and found that administration of amifostine 30 mins prior to CYP injection protected against CYP-induced loss in taste sensitivity. Morphological studies showed that pre-treatment with amifostine prevented CYP-induced reduction in the number of fungiform taste papillae and increased the number of taste buds. Immunohistochemical assays for markers of the cell cycle showed that amifostine administration prevented CYP-induced inhibition of cell proliferation and also protected against loss of mature taste cells after CYP exposure. Our results indicate that treatment of cancer patients with amifostine prior to chemotherapy may improve their sensitivity for taste stimuli and protect the taste system from the detrimental effects of chemotherapy.

## Introduction

Chemotherapy for cancer often has side effects such as alopecia, nausea, nephrotoxicity and taste alterations [1], [2]. Cyclophosphamide (CYP), a chemotherapy drug, is prescribed worldwide for various types of cancer either by itself or in combination with other drugs [3]. As a DNA-alkylating agent, CYP can damage the DNA of target cells by forming intra-strand or inter-strand cross linkages [4], [5]. Proliferating cells are especially sensitive to CYP due to conformational changes in DNA. Like many chemotherapy drugs, CYP affects healthy normal cells undergoing division as well as rapidly proliferating cancerous cells. Consequently, dividing progenitor cells and cell types with rapid turnover rates such as hair follicle cells and salivary gland cells are susceptible to the toxic effects of CYP.

Taste cells have a short renewal time of eight to twelve days [6], [7] and therefore may also be susceptible to CYP toxicity. Notably, about 50–80% of cancer patients who receive either chemotherapy or radiation-based treatment experience taste alteration in the form of reduced taste sensitivity (hypogeusia), distorted taste perception (dysgeusia), or even complete loss of taste (ageusia) during treatment [1], [8]–[11]. Moreover, these deficits can potentially last for months or years beyond the end of therapy, resulting in a critical health problem. Alterations in taste sensation can lead to reduced food intake, loss of appetite, malnutrition, lower quality of life, and slower recovery, especially in older or advanced cancer patients [1], [10], [12], [13]. Accordingly, protecting normal taste epithelium from the effects of chemotherapy drugs such as CYP may have major health benefits.

Amifostine (AMF) is a candidate drug that may protect taste. AMF is an FDA-approved sulfhydryl compound (also known as ethyol or WR2721) that is administered to some patients as a cyto-protective treatment before chemotherapy and radiotherapy. AMF protects cells by scavenging free radicals and regulating the transcription of genes involved in apoptosis, cell cycle and DNA repair [14]–[16]. It selectively protects normal cells over neoplastic cells from radiation and chemotherapy because of the relatively higher pH, alkaline phosphatase activity, and vascular permeability of normal tissue compared to cancerous tissue [17], [18]. It has been used to reduce or prevent xerostomia in patients undergoing radiation therapy for head and neck cancer [18], [19] and chemotherapy-induced cytotoxicity for a wide range of chemotherapy drugs including CYP [18]. AMF has been well-tolerated in patients with advanced malignancies and reduced CYP-induced hematological toxicities, granulocytopenia, nephrotoxicities and myelosuppression [16]. However, no reports indicate whether or not AMF can protect taste epithelium from chemotherapy-induced toxicity. Here we demonstrate that pretreatment with AMF protects the peripheral taste system from the cytotoxic effects of CYP on taste function in a mouse model. Our results may lead to improved cancer treatment strategies.

## Results

### AMF protects against CYP-induced deficits in taste sensitivity

Existing clinical literature suggests that chemotherapy drugs may elevate taste thresholds [8]. Our previous work with umami taste stimuli showed that mice experienced a two-phase elevation in MSG and IMP detection thresholds following a single IP injection of CYP [20]. Thus, it was important to see if the detection threshold of sucrose was affected in the same way as MSG and IMP after an IP injection (75 mg/kg) of CYP.

**Figure 1:**
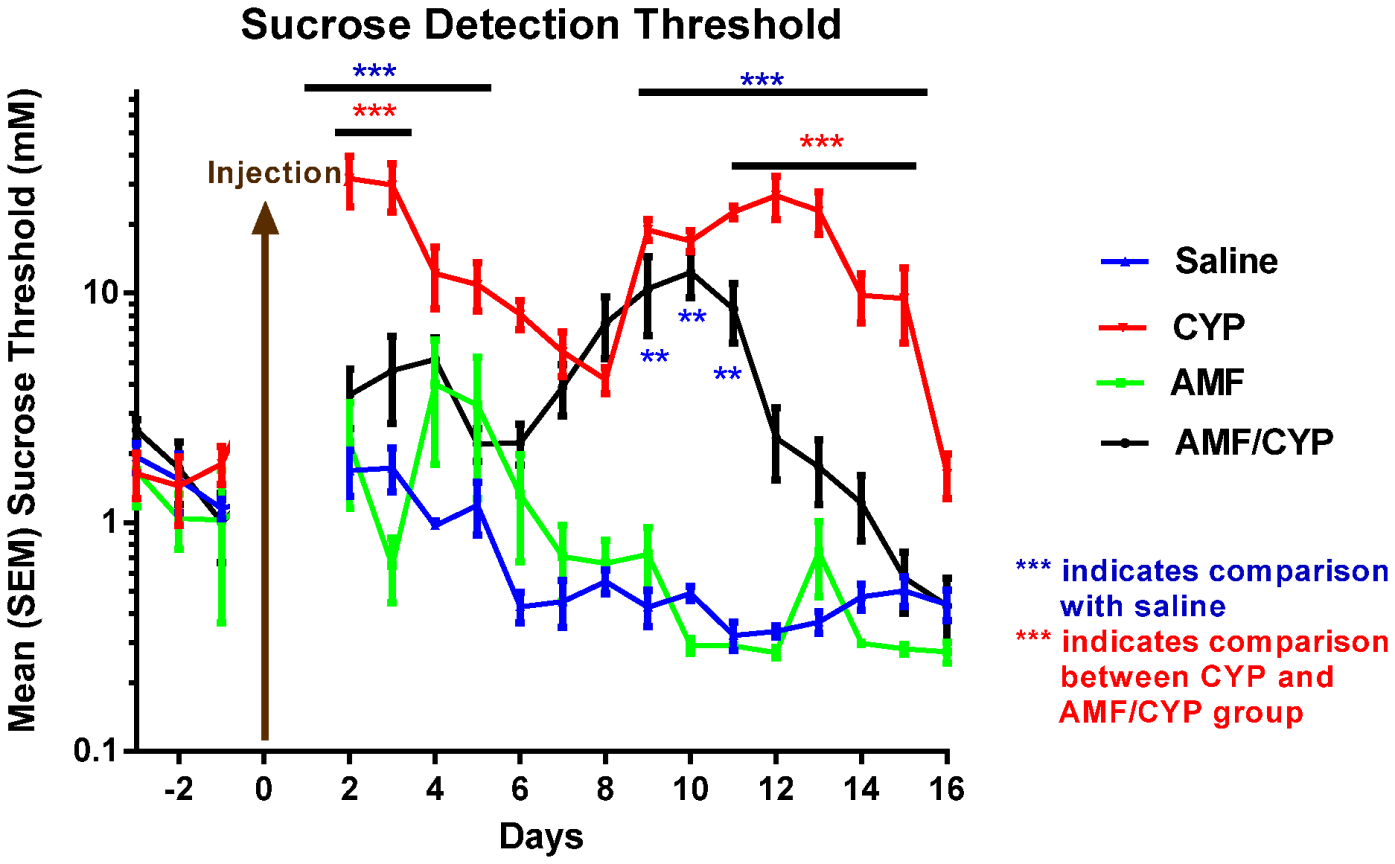
AMF protects against the CYP-induced decreases in taste sensitivity and the number of fungiform papillae with and without pore, and improves overall morphological index of fungiform taste buds. (A) Sucrose detection thresholds, before and after saline, CYP, AMF or AMF/CYP injection. The graph shows the mean (± SEM) threshold concentration of sucrose (Y-axis, log scale) across days (X-axis) post-injection. Days −1, −2 indicate thresholds before the injection. CYP-injected mice showed significant elevations of sucrose detection thresholds on days 2–5 and 9–15 after injection, while AMF/CYP-injected mice showed significant elevation in sucrose detection threshold on days 9–11 post-injection. (B) Mean (± SEM) number of all fungiform papillae across days post-injection. CYP injection significantly decreased the total number of fungiform taste papillae on post-injection days 4, 7 and 10 compared with saline control mice. AMF/CYP-injected mice had significantly higher number of papillae compared to CYP-injected mice on days 4 and 7. (C) Mean (± SEM) number of fungiform papillae with a taste pore across days post-injection. There was also a significant drop in the number of fungiform taste papillae with pores on days 4, 7 and 10 compared with saline controls. AMF/CYP groups had significantly higher number of papillae with pores on days 4, 7 and 10 compared to CYP groups. To the right of the graph are examples of a fungiform papilla without (upper image) and with a pore (lower image). The red arrow identified the opening of a pore. (D) Morphological index of fungiform papillae across days in saline-, CYP- or AMF/CYP-injected mice. The morphological index is expressed as p/P, where p = proper taste buds and P = Total number of taste buds. There was a significant decrease in the morphological index for CYP-injected mice on days 4, 7 and 10 compared to saline controls. AMF/CYP mice revealed a protective effect of AMF on days 4 and 7 (*** *P*<0.001;** *P*<0.01;* *P*<0.05).

The mean thresholds for sucrose taste detection in mice injected with saline, CYP, AMF, and AMF/CYP are shown in Figure 1a. No group differences in sucrose detection threshold were observed during the pre-injection period. In contrast, we found significant differences between treatment groups in the post-injection period. Analysis of post-injection data by repeated-measures ANOVA demonstrated a significant main effect of days post-injection [F (14, 127) = 4.700, P<0.001], treatment group [F (3, 38) = 59.381, P<0.001], and also a significant group by days interaction [F (42, 127) = 4.332, P<0.001]. There was no difference in the sucrose detection thresholds pre- and post-injection in saline-injected mice (controls). Sucrose detection thresholds in the AMF group did not change post-injection. Furthermore, sucrose detection thresholds did not differ between the AMF and saline groups post-injection. Simple effects tests showed a significant elevation in sucrose detection thresholds of CYP-injected mice compared with saline controls on days 2–5 (P<0.001) and days 9–15 post-injection (P<0.001 or less for all days). For example, sucrose thresholds for CYP-injected mice on day 4 post-injection averaged 12.196±3.692 mM (mean ± SEM) whereas the thresholds for the saline-injected mice averaged 0.971±0.041 mM. The thresholds of the CYP group returned to baseline by 16 days post injection.

The AMF/CYP-injected group had significantly elevated sucrose detection thresholds relative to the saline group on days 9–11 (day 9 and 10, P<0.01; day 11, P<0.05; Figure 1). Differences in sucrose detection thresholds between saline controls and the CYP treatment groups were greatest on post-injection day 12 (Mean threshold concentration ± SEM: saline, 0.335±0.019 mM; CYP, 26.649±5.693 mM; AMF/CYP, 2.344±0.816 mM; Figure 1). To evaluate the efficiency of AMF in preventing deficits in taste function, we compared the CYP-injected group with the AMF/CYP-injected mice. The mice in the AMF/CYP group were significantly more sensitive to sucrose than the mice of the CYP group on days 2–3 (P<0.001) and days 11–15 after CYP exposure (days 11–13, P<0.001; days 14 and 15, P<0.05; Figure 1). Collectively, the results indicated that AMF treatment provided partial protection against CYP-induced elevations in sucrose detection thresholds.

Clinical studies have revealed that patients undergoing chemotherapy often experiences significant weight loss [21]. Consequently, we carefully monitored the body weights for all four groups of mice (saline, AMF, CYP, AMF/CYP) throughout the training and testing stages to see whether the changes in taste sensitivity were due to changes in motivation. The body weights of all groups were comparable throughout the experiment and no drug related changes in weight in any of the groups during post-injection days could be detected. We also compared the daily number of trials completed by the mice in each group. Neither the AMF-only or AMF/CYP group showed any significant decrease in the number of trials completed each day compared to saline-injected control mice. The CYP-injected mice completed significantly fewer trials only on day 2 post-injection compared with the saline-injected mice (P<0.5). The mean +/− SEM number of trials completed on day 2 by saline-injected group was 90 +/−9, 55+/−11 by CYP-injected mice, 84 +/−4 by AMF-injected mice, and 88+/−7 by AMF/CYP-injected mice. Thereafter, mice in all groups typically completed between 90–110 trials per session.

### AMF protects against CYP-mediated destruction of fungiform taste papillae

**Figure 2:**
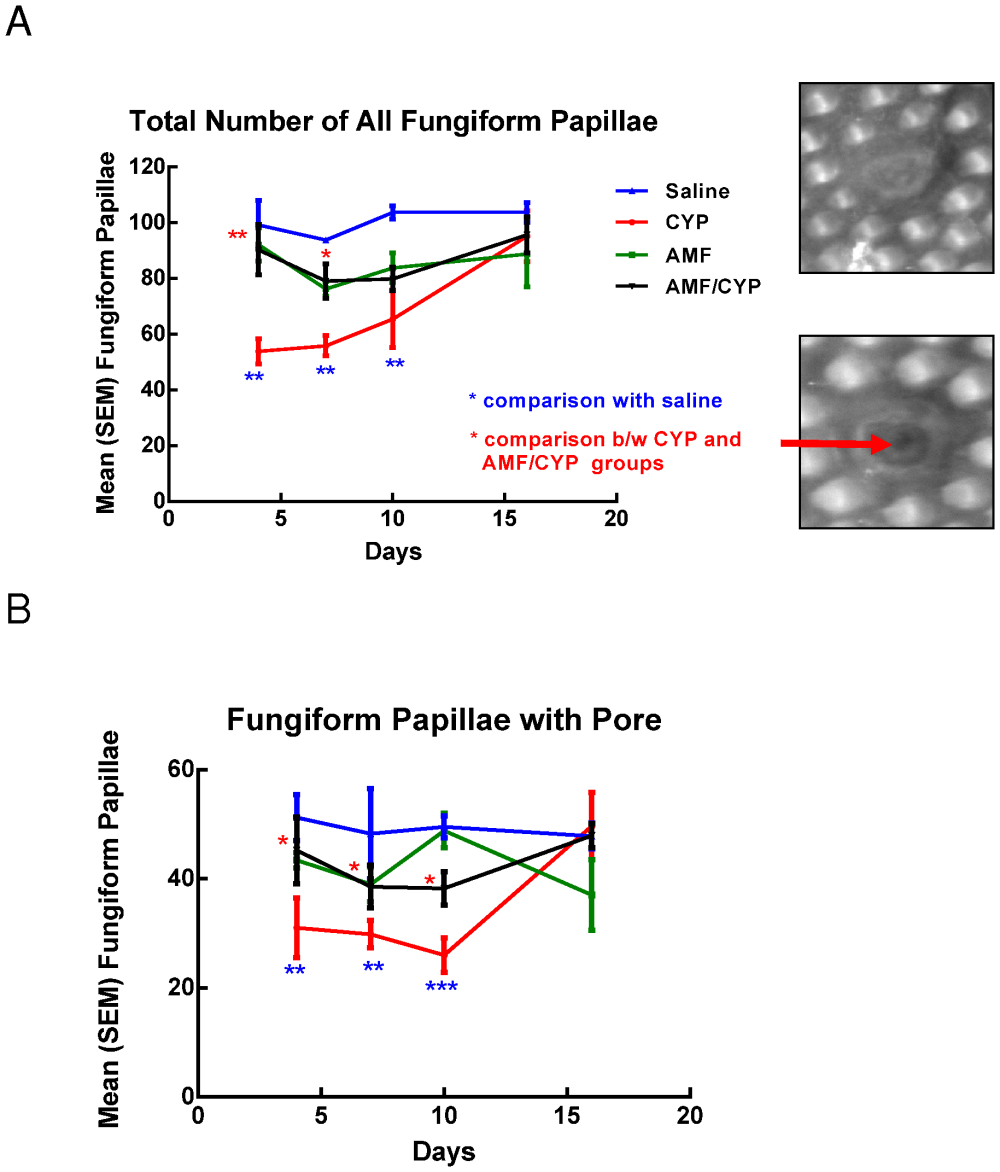
Representative bright field images of H and E stained fungiform and circumvallate taste buds at Days 4, 7, 10 and 16 post-injection of saline, CYP or AMF/CYP-injected mice. (a) Images of fungiform taste buds. Morphologically intact taste buds with organized mass of taste cells (indicated by black arrow) are abundant in saline-injected (control) mice. There was a disruption in the organization of taste buds, seen as an absence of organized mass of taste cells (indicated by green arrow) in CYP-injected mice on day 4 which does not recover until day 16 post-injection. AMF/CYP-injected mice do not show this disruption except on day 7. (b) Trench of a circumvallate papilla with taste buds. The red-boxed area shows the region which is magnified to observe detailed morphology. (c) Images of circumvallate taste buds. There was no morphological change in the circumvallate taste buds of saline and AMF mice across days. However there were open-spaces (red arrow) inside the taste buds on days 7 and 10 in CYP-injected mice. AMF/CYP–injected mice showed such “open spaces” (red arrow) only on day 7. Scale bar = 25 µm.

Our behavioral data revealed two periods of taste deficits after CYP administration, therefore we examined the lingual epithelium for potential changes that might correspond to the observed disruptions in taste function. Compared with saline controls, the total number of fungiform papillae (with or without pores) was sharply lower at day 4 after CYP injection and did not recover until day 16 after injection (Figure 2A). Comparison of the number of papillae in saline control (day 0) and CYP-injected groups (days 4, 7, 10, and 16) indicated significant differences between the drug groups [F (3, 15) = 11.592, P<0.001] and days [F (3, 15) = 5.208, P<0.01]. Post hoc comparisons indicated that the number of papillae on the tongues of saline-injected mice was significantly greater than the papillae on CYP-injected mice (P<0.01). However, the number in saline-injected mice did not differ from that of AMF- or AMF/CYP-injected mice (Dunnett's test). The data were then partitioned to compare the effect across days. Compared with saline controls, we found that the number of papillae in the CYP condition decreased on day 4 (P<0.01), and remained reduced on days 7 and 10 (P<0.01 for both days). The number of papillae in CYP-injected mice returned to normal levels on day 16. The AMF/CYP groups had a significantly higher number of taste papillae on days 4 and 7 compared with the CYP groups (P<0.01 on day 4; P<0.05 on day 7). Notably, the number of fungiform taste papillae in the AMF/CYP groups did not differ from that of saline controls or mice treated with AMF alone (Dunnett's test), indicating protection by AMF treatment.

In post-natal mice, histological studies reveal approximately 32% of fungiform taste papillae are associated with a taste pore and it is generally accepted that a pore is necessary for the taste bud to be functional [22]–[25]. Compared with saline controls, the number of taste papillae with a taste pore decreased in CYP-injected mice and did not recover until 16 days after injection [drug group comparison: F (3, 15) = 10.701, P<0.001; days: F (3, 15) = 2.649, P = <0.05]. This decrease was not seen in other groups (Figure 2B). The CYP groups significantly differed from the saline (control) groups at days 4, 7, and 10, but returned to normal at day 16 (P<0.01 on day 4 and 7 ; P<0.001 on day 10). The AMF/CYP groups had a significantly higher number of papillae with pores on days 4, 7 and 10 compared with the CYP groups (P<0.05 on all days).

### AMF improves morphological index of fungiform taste buds

**Figure 3:**
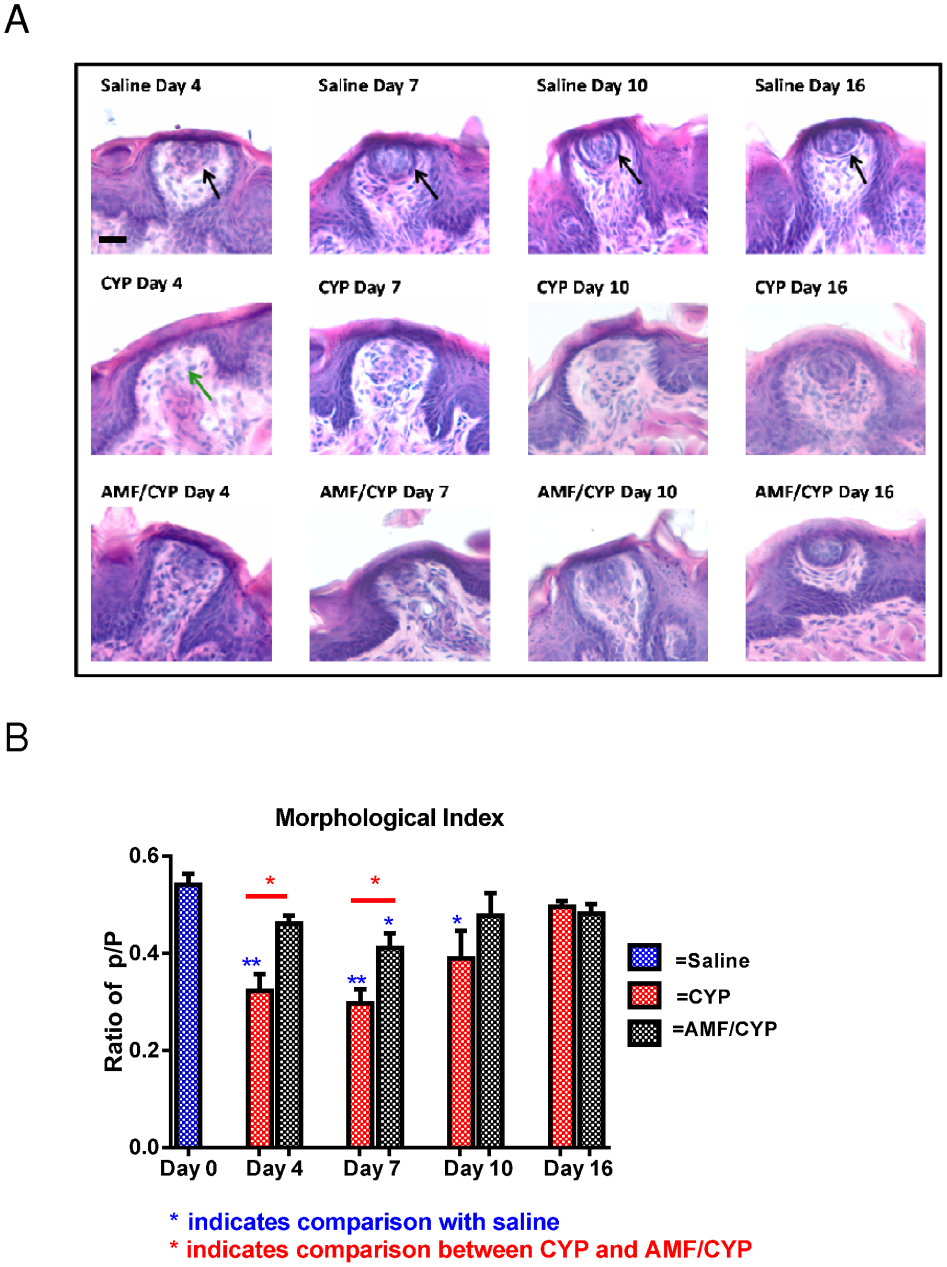
Mean (± SEM) number of BrdU-positive cells in the basal layer of fungiform papillae and taste buds, one wall of each circumvallate trench, and a sample area of the lingual epithelium in Saline, CYP and AMF/CYP mice on days 4, 7, 10 and 16 post–injection. (**a**) BrdU-positive cells in the basal layer of fungiform taste papillae and taste buds of each drug condition. (**b**) BrdU-positive cells in the basal layer of one wall of a circumvallate trench for each drug condition. (**c**) BrdU-positive cells in lingual epithelium of each drug condition (*** *P*<0.001;** *P*<0.01;* *P*<0.05).

To examine further CYP-induced disruption of taste buds, harvested tongues were sectioned and stained with hematoxylin and eosin to examine fungiform and circumvallate taste buds. In addition to a significant decrease in the number of fungiform taste buds, we also observed disorganization of cells inside the remaining taste buds of CYP-injected mice (Figure 3A). The organization of taste cells in the central third of a fungiform papilla, previously observed in ultrastructural studies of rodents [24]–[27] was used as a criterion to define a morphological index for fungiform taste buds [24], [25], [27]. The presence of a well-organized mass of cells was judged as an intact taste bud whereas the absence of apparent organization was considered an incomplete taste bud [24]. The index for intact taste buds was calculated as a ratio of p/P where p = the number of normal taste buds with a well-defined full complement of cells (as indicated in saline-injected mice of Figure 3A), and P = the total number of taste buds (both normal and incomplete taste buds lacking cells). The p/P ratio was intended to determine whether fungiform papillae had recognizable taste buds and was used to compare the morphology of fungiform taste buds across days and groups (Figure 3B). The morphological index of taste buds decreased in CYP-injected mice on days 4, 7 and 10 but not in AMF/CYP-injected mice or AMF only controls. For example, the morphological index of fungiform taste buds was 0.54+/−0.05 in saline injected mice and 0.46+/−0.015 in day 4 AMF/CYP-injected mice. However, the ratio dropped to 0.32+/−0.03 in day 4 CYP-injected mice. There was a significant difference between drug groups across days post-injection [F (12, 50) = 5.336, P<0.001]. The morphological index was significantly greater in saline-injected mice than CYP-injected mice on days 4, 7 and 10 (P<0.01 on days 4 and 7; P<0.05 on day 10, Figure 3B), but did not differ from the AMF-injected mice. The morphological index for AMF/CYP-injected mice was significantly lower than saline-injected mice only on day 7 (P<0.05). However, the morphological index of AMF/CYP-injected mice was significantly higher on days 4 and 7 compared with CYP-injected mice (P<0.05 for both days, Figure 3B), indicating that the proportion of intact taste buds was significantly greater in the mice injected with AMF prior to the CYP injection compared to those mice receiving only the CYP injection.

**Figure 4:**
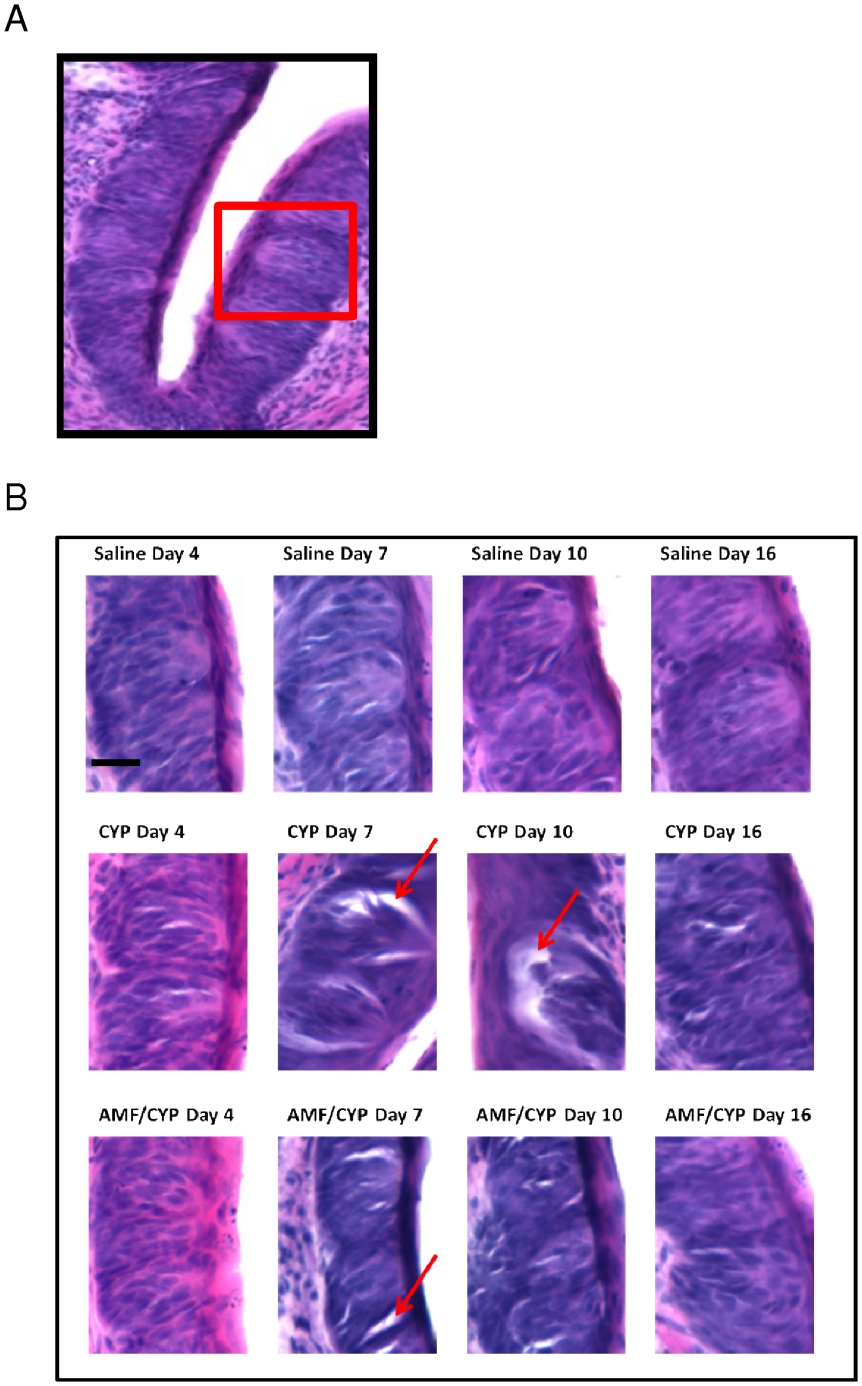
Representative images of Ki67-positive cells (red) in fungiform taste papillae with taste buds and in taste buds in circumvallate trenches on days 4, 7, 10 and 16 post-injection of saline-, CYP- or AMF/CYP-injected mice. Sytox green was used as a nuclear marker. There was a reduction in the number of Ki67-positive cells in the basal layer of fungiform taste papillae and the basal layer of circumvallate taste buds on day 4 in CYP-injected mice but not in AMF/CYP-injected mice. The bar graphs illustrate the mean (± SEM) number of Ki67-positive cells in fungiform papillae (upper graph) and the basal layer of one wall of each circumvallate trench (lower graph) across days in each drug group. (**a**) Ki67-positive cells in the basal layer of fungiform taste papilla and taste bud. Scale bar = 25 µm. (**b**) Ki67-positive cells in the basal layer of one wall of each circumvallate trench. Scale bar = 50 µm (*** *P*<0.001;** *P*<0.01;* *P*<0.05).

Detectable drug-induced changes in circumvallate papillae appeared to follow a different temporal pattern. Comparison of hematoxylin and eosin-stained sections of taste buds of circumvallate papillae did not reveal observable differences between groups on day 4 (Figure 4). However, at later time points there were apparent “open spaces” in taste buds of the CYP group, indicating missing cells. To evaluate these observations, Image J was used to compare the area of open space in these taste buds. The average maximum area observed between adjacent taste cells in each of the saline control tongues (n = 5) was averaged, and then the average was used as a baseline value. If the area between adjacent cells was greater than the baseline value, the area was considered as “open space”. In CYP groups, “open spaces” in taste buds were readily detected on days 7 and 10, similar to effects of CYP on circumvallate taste buds described in a previous study [20]. Of note, we observed little or no open spaces in the taste buds of saline control mice or in the mice that received AMF alone (AMF groups). In the AMF/CYP groups we observed taste buds with open spaces on day 7, but not on day 10. A simple effects test comparing the open space measures of CYP- and AMF/CYP-injected mice at days 7 and 10, and the day 0 mice revealed detectable differences between groups across days [F (8, 36) = 9.183, P<0.001]. Post hoc testing found the “open spaces” in the CYP-injected groups were significantly greater than those of saline control mice on day 7 (P<0.01) and day 10 (P<0.001) and AMF/CYP group on day 7 (P<0.01).

### AMF protects against CYP-induced inhibition of cell proliferation

**Figure 5:**
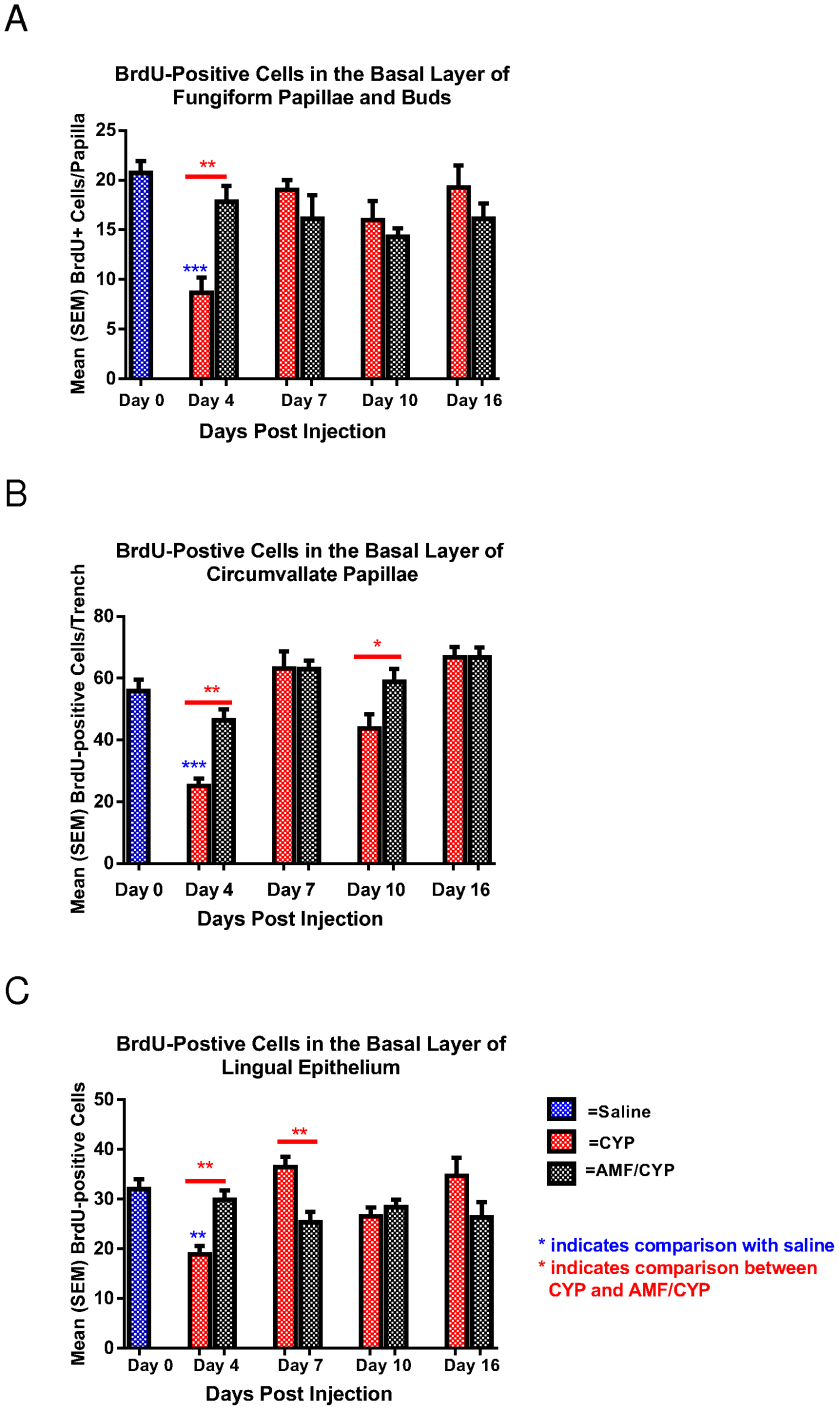
Representative images of PLCβ2-positive cells (red) in fungiform and circumvallate taste buds on days 4, 7, 10 and 16 post-injection in saline-, CYP- or AMF/CYP-injected mice. Sytox green was used as a nuclear marker. There was a reduction in the number of PLCβ2-positive cells in fungiform and circumvallate taste buds on day 4 in CYP-injected mice but not in AMF/CYP-injected mice. The bar graphs illustrate the mean (± SEM) number of PLCβ2-positive cells in fungiform (upper graph) and circumvallate (lower graph) taste buds across days in each drug group. (**a**) In fungiform taste buds, there was a reduction in the number of PLCβ2-positive cells on day 4, 7 and 10 in CYP-injected mice and on day 7 in AMF/CYP-injected mice. Scale bar = 25 µm. (**b**) In circumvallate taste buds, there was a reduction in the number of PLCβ2-positive cells on day 7 and 10 in CYP-injected mice and on day 7 in AMF/CYP-injected mice. Scale bar = 50 µm. (*** *P*<0.001;** *P*<0.01;* *P*<0.05).

BrdU, a thymidine analogue, is commonly used in the detection of cell proliferation [28]. BrdU is incorporated into DNA during the S-phase of the cell-cycle. We used BrdU as a marker to study cell proliferation in the basal layer of taste papillae, taste buds and lingual epithelium. We first examined the basal layer of both fungiform papillae as well taste buds, and observed a reduction in the number of BrdU-labeled cells on day 4 post-injection in CYP-injected mice but not in AMF/CYP-injected mice (Figure S1A). The observation was confirmed by statistical analysis of BrdU-positive cell counts [F (12, 68) = 6.794, P<0.001; Figure 5A]. Four days after CYP injection, the number of BrdU-positive cells in the CYP group was significantly less than the number in saline controls (P<0.001, Figure 5A). By contrast, the number of BrdU-positive cells in AMF/CYP-injected mice did not differ from that in saline controls or AMF-injected mice at any time point examined (Figure 5A). There were significantly more BrdU-labeled cells in fungiform papillae of the AMF/CYP group compared with the CYP group on day 4 after injection (P<0.01), indicating that AMF protected at least some of the cells that proliferate during normal cellular turn-over of the taste bud from the toxic effects of CYP.

The number of BrdU-positive cells in the circumvallate papillae, like the fungiform papillae, also decreased in response to CYP injection [F (12, 65) = 8.459, P<0.001]. There was a significant reduction in the number of proliferating cells 4 days after CYP injection relative to the number in saline controls (P<0.001). In contrast, a decrease in BrdU-labeled cells was not observed in mice treated with AMF before CYP (Figure 5B and Figure S1B).

The AMF/CYP groups had a significantly higher number of proliferating cells in the circumvallate papillae compared with the CYP groups on day 4 (P<0.01) and 10 (P<0.05) after CYP injection (Figure 5B).

The number of BrdU-labeled cells in the non-taste lingual epithelium was also evaluated. This analysis showed a similar pattern in response to CYP and AMF as that observed in the fungiform and circumvallate papillae. The comparison of CYP-injected mice to AMF/CYP-injected mice demonstrated a significant protective effect of AMF treatment on proliferating cells on day 4 after CYP injection (P<0.01, Figure 5C and Figure S1C). The significant elevation of BrdU-positive cells (P<0.01) in the CYP group compared with the AMF/CYP group on day 7 indicated a rebound effect on non-lingual cell proliferation following CYP injection.

**Figure 6:**
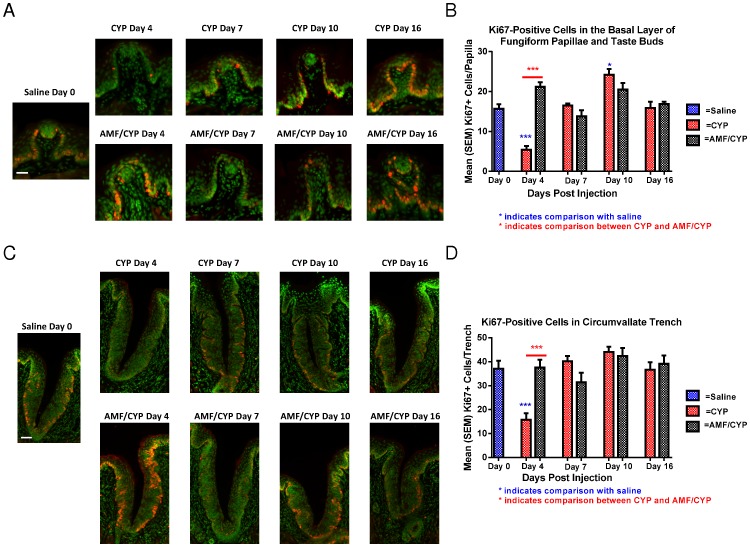
Ki67-positive cells.

To verify the effects of CYP and AMF on cell proliferation, we stained tissue sections for Ki67, a marker that labels all but G_0_ phase of cell cycle activity. We examined the number of Ki67-positive cells in the basal layer of both the fungiform papillae and taste buds. There was no difference in the number of Ki67-positive cells between saline controls and AMF-injected mice at any time point examined. However, there were differences in the number of Ki67-positive cells between the saline control and CYP-injected mice across days [F (12, 56) = 10.830, P<0.001, Figure 6A and 6B]. Compared to saline control mice, there was a decrease in the number of Ki67-positive cells in fungiform taste buds of CYP-injected mice on day 4 (P<0.001), followed by an increase on day 7 and 10 (Figure 6A and 6B). In CYP-treated mice, the number of Ki67-positive cells exceeded the number observed in saline controls at day 10 (P<0.05), and returned to control levels by day 16 (Figure 6A and 6B). AMF treatment prior to CYP exposure prevented the decrease in Ki67-positive cells at day 4 (P<0.001) and also the rise above control numbers at day 10 (Figure 6A and 6B). These data also suggest a rebound effect in cell proliferation in the CYP-injected mice.

In the case of circumvallate taste buds, there was a similar pattern of change in the number of Ki67-positive cells [F (12, 56) = 5.576, P<0.001, Figure 6C and 6D]. There was no difference in the number of Ki67-positive cells between the saline-injected (control) and AMF-injected mice. Compared with controls, the number of Ki67-positive cells in CYP-injected mice decreased at day 4 (P<0.001), and returned to normal numbers on day 16 (Figure 6C and 6D). In contrast, at day 4 the number of Ki67-positive cells in AMF/CYP mice did not decrease relative to saline controls (Figure 6C and 6D). The comparison between the CYP-injected and AMF/CYP-injected mice indicated that AMF also protected against the CYP-induced decrease in the number of Ki67-positive cells on day 4 in circumvallate papillae (P<0.001, Figure 6C and 6D).

### AMF partially protects against CYP-induced loss of mature taste cells

A taste bud is comprised of several cell types important for normal taste functioning. Type II (receptor) cells are G-protein coupled receptor cells that detect bitter, sweet or umami [29]. These cells utilize a second messenger transduction pathway in which PLCβ2 is a key component of the signaling pathway [30]. Since CYP and AMF treatments altered taste sensitivity to sucrose, we examined potential post-injection changes in Type II cells using PLCβ2 as a marker.

**Figure 7:**
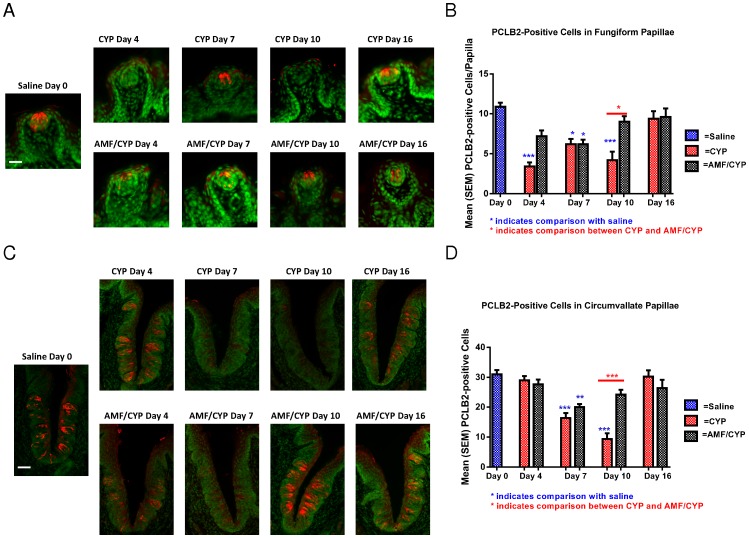
PLCβ2-positive cells.

We observed a two-phase loss of PLCβ2-positive cells after CYP injection compared with staining patterns in saline controls (Figure 7A and 7B) in fungiform papillae, suggesting an apparent biphasic decrease in Type II cells following CYP administration. There was no difference in the mean number of PLCβ2-positive cells per fungiform taste bud profile between the saline controls and AMF-injected mice at any time point. In contrast with control mice, the number of PLCβ2-positive cells in CYP-injected mice decreased on days 4, 7 and 10 after CYP injection (P<0.001 on days 4 and 10, and P<0.05 on day 7), returning to control levels by day 16 (Figure 7A and 7B). In the case of AMF/CYP group, there was a decrease in the number of PLCβ2-positive cells on day 7 (P<0.05, Figure 7A and 7B). By day 10 after CYP injection, the number of PLCβ2-positive cells in fungiform taste buds of AMF/CYP group was similar to that in saline controls and significantly higher than that in CYP-injected mice (P<0.05, Figure 7A and 7B). Thus AMF partially protected Type II cells in fungiform taste buds from the second phase of taste disturbance.

In terms of PLCβ2 staining, the circumvallate papillae were affected by CYP and AMF in a different manner than the fungiform papillae. At day 4 after injection, there was no reduction in the mean number of PLCβ2-positive cells per circumvallate taste bud profile in the CYP group compared with saline controls. However, there was a reduction in the number of PLCβ2-positive cells on days 7 and 10 after CYP injection (P<0.001), with a return to control cell numbers by day 16 (Figure 7C and 7D). In the circumvallate taste buds of the AMF/CYP mice, there was a decrease in the number of PLCβ2-positive cells at day 7 compared with controls (P<0.01) but not at day 10 (Figure 7C and 7D). The number of PLCβ2-positive cells in the AMF/CYP group was significantly higher than that in the CYP group at day 10 (P<0.001; Figure 7C and 7D). There was no difference in the number of PLCβ2-positive cells between saline controls and AMF-injected mice at any time point examined.

## Discussion

Using a mouse model, we previously demonstrated a two-phase disruption in umami taste function following a single IP injection of CYP [20]. The first phase of taste disruption occurred at 2–4 days and the second phase occurred at 9–12 days after CYP-injection. Further evidence suggested that the first phase may be due to the cytotoxic effects of CYP on fungiform papillae, taste buds, von-Ebner glands, and motivational status of mice, while the second phase may be due to the disturbance in taste cell kinetics [20]. In the present work, we determined whether the cytotoxic effects of CYP on taste function could be alleviated by AMF treatment. To our knowledge, this is the first report to test the potential of AMF in reducing CYP-induced taste deficits. Our results indicate that pre-treatment with AMF ameliorated CYP-induced taste deficits by reducing the loss of fungiform taste papillae, increasing the number of morphologically-intact taste buds (morphological index), reducing CYP-induced inhibition of cell proliferation, and protecting against loss of mature taste cells after CYP exposure.

In preclinical studies, AMF (200–400 mg/kg, s.c.) showed significant protection of hematopoietic progenitor cells from a broad range of chemotherapeutics, including CYP. AMF was found to be effective in reducing CYP-induced hematological toxicities in both *ex vivo* and *in vivo* studies [31], [32]. When administered one hour prior to irradiation, AMF (200 mg/kg, s.c.) provided complete protection against radiation-induced mucositis in rat [33]. Similarly, it was shown to protect rat salivary gland, oral mucosa, and hair follicles from the side effects of irradiation [34], [35]. In addition, AMF injection (200 mg/kg, i.p.) protected against cisplatin-induced nephrotoxicity [36]–[38] and CYP-induced pulmonary toxicity [39].

The encouraging results in pre-clinical studies led to clinical trials to test the efficacy of drug after its approval by the FDA in 1996 [17]. Phase I clinical trials indicated that AMF was well-tolerated in patients with advanced malignancies, and that it reduced CYP-induced hematological toxicities [40], [41]. Phase II clinical trials showed that AMF reduced both the degree and duration of granulocytopenia during CYP therapy [42]. Phase III clinical trials have found AMF to be protective against CYP and cisplatin-induced hematological toxicity and neutropenia [43], nephrotoxicities [44], and cyclophosphamide-induced myelo-suppression [45].

Thus, an extensive amount of work has been done towards evaluating the effects of AMF in cancer treatment, but its efficacy has never been tested in chemotherapy drug-induced taste disorders. Our results indicate that AMF provides at least partial protection again CYP-induced taste deficits. Notably, compared with many of the studies cited above, we used a dose of 100 mg/kg, half the dose used in other studies that reported complete protection. Although the drug is well-tolerated, its use comes with dose-dependent side effects of hypotension, nausea, metallic taste and asthenia [46]. Since emesis and metallic taste can influence taste functions, we chose a lower dose of AMF to minimize these side effects and avoid their confounding effects on taste. At the higher doses typically used in clinical applications, we would expect AMF to provide even more protection for the taste system.

The effects of CYP or other chemotherapy drugs on thresholds for basic tastes of patients have been obtained primarily through clinical interviews or self-report. One of the larger scale clinical studies reported that out of 518 patients undergoing a chemotherapy regime, 347 (67%) self-reported changes in taste. Of these, changes in salt (41%) and sweet (36%) tastes were reported more often than sour (21%) or bitter (24%), indicating that some tastes may be more susceptible to chemotherapy drugs than others [12]. Another report indicated that patients experience decreased sensitivity for sweet and sour tastes, but increased sensitivity for bitter [47], [48]. However, the effects of chemotherapy drugs need to be assessed in clinical populations with more rigorous psychophysical methods. Based on our findings, we hypothesize that all taste modalities will be affected by CYP administration. We would predict that thresholds would be elevated for all basic tastes, except possibly bitter. Ongoing research in our laboratory is exploring whether all the subtypes of taste cells are being affected in the same way.

The biphasic temporal pattern of taste disruption observed in this study is not predicted by learning theory or by CTA because of our experimental design. Optimal conditioned taste aversion learning occurs when the taste of a novel stimulus is temporally proximal to the sensations of an upset stomach [49]. In this and our previous experiments, the CYP injection was administered almost 24 hours after the last presentation of very familiar taste stimuli, thus preventing the temporal contiguity between nausea and a familiar taste stimulus. In addition, we routinely monitor several measures i.e., body weight and number of trials/session, for motivational changes related to drug injections. There was no significant difference in the weight of the mice across days in any of the groups in the pre- or post-injection days and the trials per day were adversely affected by CYP only the first day (day 2) after the mice were restarted on the threshold study compared with the saline-injected (control) mice. Collectively these data suggest that even though motivational levels may have been affected within 1–2 days after injection, motivational levels were similar for the rest of the experiment and confirm our previous report [20]. Even so, the AMF-only or AMF/CYP group did not show any significant decrease in the number of trials compared to control mice. With regard to other modalities, we do not have evidence from these experiments whether CYP has a direct effect on the olfactory system. This study also does not address the issue of whether CYP can have direct effects on higher levels of the taste system within the central nervous system. However, this possibility would seem unlikely as none of the central structures within the taste system is known to have a cell population highly susceptible to the molecular effects of CYP.

We found that when proliferation resumes in taste epithelium (e.g., as indicated by Ki67-positive cells), the proportion of cells in S phase (BrdU-positive cells) increases to levels significantly higher than controls. This observation is comparable to the finding of Nguyen et al. [50] where they found a rebound effect in the taste epithelium after irradiation. This result is similar to the regenerative response of other tissues (like skin and gut) to irradiation. The increased numbers of proliferative cells are either due to production of additional taste bud stem cells via symmetric divisions, and/or a shortened cell cycle of transit amplifying cells [51] to make up for the cell cycle arrest.

Interestingly, our data indicate that fungiform and circumvallate taste papillae are differentially sensitive to the effects of CYP. Fungiform taste papillae are affected immediately after CYP injection on day 4 while the circumvallate papillae are not affected until day 8 post-CYP injection. However the reason behind the greater susceptibility of fungiform taste papillae remains elusive. One possible reason may be the anatomical location of the fungiform taste buds on the surface of the tongue. It is possible that metabolites of CYP might have entered the mouth with crevicular fluid. This fluid originates in the plasma and come to the mouth during chewing [1]. Since the fungiform papillae are more exposed on the surface of the tongue and are located more proximal to the gum, they might be a more susceptible target than the circumvallate. Another potential reason might be related to the differential configuration of galactosyl residues in the two types of taste buds and the presence of mannose in circumvallate taste buds but not in fungiform taste buds [52] . It is also possible that the mucins and N-linked glycoproteins (with a hormone-like paraneuronal function) [52] present in the circumvallate and not in fungiform provide protective effects against cytotoxicity in circumvallate taste buds. Other potential explanations for the differences in susceptibility to CYP between circumvallate and fungiform papillae may be due to differences in their embryonic origins [53], or to possible differences in vascularization [25]. Nguyen and Barlow [54] have also found differential expression of BMP4 in fungiform and circumvallate taste buds of adult mice and thus may play an important role in response to cytotoxic challenges. In addition, the taste pore keratinocytes, with a fast renewal rate, are highly affected by the cytotoxicity of CYP. This might be one of the causes of the sharp drop in the number of fungiform papillae both with and without pores on day 4. All these reports indicate that there are subtle differences between the two kinds of taste papillae and one or more of these factors may contribute to the differential effects of CYP. The reasons for this interesting difference in CYP-susceptibility are currently being explored.

In summary, our results indicate that the deficits in taste functions observed in these experiments are mostly due to the cytotoxic effects of CYP and disruptions in the taste cell replacement cycle. The ability of AMF to protect taste function after CYP exposure is promising in terms of future design of treatment strategies for cancer patients. Further experiments could examine a dose-response relationship to determine if AMF is able to provide complete rather than partial protect against taste deficit after chemotherapy.

## Materials and Methods

### Subjects

Male C57BL/6J mice, obtained from Jackson Laboratory (Bar Harbor, ME), were housed in groups of 2–4 mice. They were at least 2 months old and typically weighed between 25–30 gm at the start of the experiment. The mouse colony was maintained on a 12/12 hr light-dark cycle with lights turned on at 7 a.m. Purina Mouse Chow (Prolab RMH 3000) and water (except in the behavioral experiment) were available *ad libitum*. The mice in behavioral experiments were adapted to a 22-hr water deprivation schedule beginning one week prior to the start of training. These mice were tested at the same time each day between 9 a.m.-2 p.m. All procedures were approved by the Institutional Animal Care and Use Committee at the University of Vermont.

### Chemical reagents

CYP (Cyclophosphamide monohydrate, 97%) was obtained from Acros Organics (New Jersey, USA). AMF [Amifostine-2-(3-Aminopropyl) aminoethyl phosphorothioate, 97%] was obtained from Sigma-Aldrich (Missouri, USA). All taste solutions were prepared daily with Millipore-filtered water (Millipore, Billerica, MA, USA).

### Behavioral test

The detection threshold experiment was carried out in five computer-controlled gustometers (Knosys Inc., Lutz, FL, USA) fitted in individual bench top stations and followed procedures used previously [20]. Briefly, 19 (5 saline-injected, 5 CYP-injected, 4 AMF-injected, and 5 AMF/CYP-injected) mice were used to evaluate sucrose thresholds by training them to responding differentially when they detected water (S+) or several concentrations of sucrose (S−). Details of the procedure are described in our previous paper [20]. Once sucrose detection thresholds were established, all animals were rehydrated and 24 hrs later the animals were injected with the assigned drugs. After another 24 hrs, all mice were returned to the water deprivation schedule. Threshold testing of mice in all groups resumed the next day and continued until 16 days after injection. Detection thresholds, determined each day, were defined as minimum concentration at which a mouse can detect sucrose at least 50% of the time on that day.

### Morphological analysis

The cellular morphology of taste papillae and taste buds of the tongue were examined at days 0 (no injection), 4, 7, 10, and 16 after the assigned drug injection. All perfusions were done with PBS-heparin followed by 4% paraformaldehyde (Electron Microscopy Sciences, PA USA) in PBS and the tongues were harvested for analysis. Details of the protocol are described in the previous published work of the lab [20]. Briefly, after fixation of the tongues for 4 hrs at room temperature, the tongues were washed with PBS, and the water soluble dye fast green was used as a stain to observe and count the number of papillae with and without pores using a dissecting microscope. The tongue was first laid flat and all the visible papillae were counted. A cactus-thorn was inserted in the non-gustatory region just posterior to the last papilla that was counted. Then the tongue was placed upright to count the number of papillae on tip, and thorns were used to mark the counted region. The papillae at the back and side of the tongue were counted in the same manner and cactus thorns were again used to delineate the boundaries and avoid double counting. All counting was done by two observers and both were blind to the injection conditions. To ensure that this method enabled the observers to correctly identify papillae with and without pores, papillae were identified and marked with cactus thorns, then frozen-sectioned to verify their status with hemotoxylin and eosin (Richa Chemical Co., Arlington, TX). For each drug condition, a total of six mice per time point were evaluated for fungiform papillae with and without pores at days 0, 4, 7, 10, and 16 post injections.

Following this procedure, each tongue was cryo-protected with a gradient of sucrose solutions (0.5–1.5 M) and cryo-sectioned at a thickness of 20 µm. Every third section was selected from the anterior 1 mm of the tongue and then stained with hematoxylin and eosin to look at the morphology of the tissue (see our previous publication [20] for a detailed protocol). Every third section from the caudal part of the tongue starting from anterior foliate papillae was stained to examine the morphology of circumvallate taste buds. Fungiform or circumvallate taste buds were evaluated only if the section was obviously of the central third of the papilla. Image J was used to evaluate the details of the taste buds.

### Cell proliferation study

BrdU-labeling was used to identify the proliferating pool of cells in the lingual epithelium, fungiform and circumvallate taste buds. For this, the mice were injected twice with BrdU (5-Bromo-2′-deoxyuridine, Sigma-Aldrich, Missouri, USA). The first injection was given at 24 hrs prior to perfusion and a second injection was given 18 hrs prior to perfusion. The tongue was harvested and sectioned as described above. Tissue processing was done on slide. The primary monoclonal antibody was diluted to 1∶500 (catalog # G3G4, Developmental Studies Hybridoma Bank) and applied to the tissue overnight at 4°C. The secondary antibody (biotinylated-anti-Mouse IgG) was used at 1∶1000 dilution (Vector Labs, California, USA) and the signal was amplified with AB solution (ABC Kit, Vector Labs, California, USA). The signal was visualized using nickel-intensified DAB (Diaminobenzidine Kit, Vector Labs, California, USA). All slides were counterstained lightly with hematoxylin as a nuclear marker.

For Ki67-labeling, the Ki67 primary antibody (SP6, Thermo Scientific) was used at 1∶200 dilution for incubation overnight at 4°C. The secondary antibody used was Alexa 546 goat anti-rabbit at 1∶1000 dilution and the incubation was done at room temperature for 2 hrs. All the slides were counterstained with Sytox green (S7020, Molecular Probes) as a nuclear marker.

### Immunohistochemistry for PLCβ2-labeling

PLC β2 primary antibody (H-255, Santa Cruz Biotechnology) was used at 1∶200 dilution for incubation overnight at 4°C and the secondary was Alexa 546 goat anti-rabbit at 1∶1000 dilution. All the slides were counterstained with Sytox green (S7020, Molecular Probes) as a nuclear marker.

### Statistical analysis

The behavioral data were analyzed by Repeated Measures ANOVA followed by simple effect tests on threshold data for each day. The papillae count data were analyzed by a two-way ANOVA followed by simple effect tests on each group and day. The BrdU-, Ki67- and PLCβ2-positive cell counts were analyzed by ANOVA procedures followed by post-hoc simple effect tests constructed such that each group and day combination was analyzed as a separate treatment group.

All statistical analysis and figures were done using SPSS software (IBM SPSS Statistics, Version 19, IBM Corporation, Chicago, IL) or Graph Pad Prism software, Version 5.0 (Graphpad software Inc., La Jolla,CA, USA).

### Image analysis

The images were captured using a Nikon Eclipse E600 Scope fitted with a color camera (Spot RT KE Diagnostic Instruments Inc.) and Spot acquisition (Spot Advanced, Version 4.6) software.

For cell proliferation studies, basal cells for all treatment conditions and on all days were counted (identified and quantified via hematoxylin or Sytox labeling) and analyzed to see whether there was any difference in total number of cells. No significant difference was found between any of the treatment groups across days.

## Supporting Information

Figure S1 **Representative images of BrdU-labeled-cells in different areas of tongue on days 4, 7, 10 and 16 post-injection in saline-, CYP- or AMF/CYP- injected mice.** BrdU-positive cells are stained in black (indicated by red arrow) and the sections are counterstained lightly with hematoxylin. Any brown staining outside the trench is non-specific staining. There was a reduction in the number of BrdU-positive cells in fungiform taste buds, circumvallate taste papillae and lingual epithelium on day 4 in CYP-injected mice but not in AMF/CYP-injected mice. (**a**) BrdU-positive cells in fungiform taste buds (indicated by red arrow). Scale bar = 25 µm. (**b**) BrdU-positive cells in circumvallate taste papillae (indicated by red arrow). Scale bar = 50 µm. (**c**) BrdU-positive cells in lingual epithelium (indicated by red arrow). Scale bar = 50 µm.
(PDF)
