# Correction: Pre-Treatment with Amifostine Protects against Cyclophosphamide-Induced Disruption of Taste in Mice

**DOI:** 10.1371/annotation/5487e265-8175-47cb-b9a4-d85862a4a96f

**Published:** 2013-07-31

**Authors:** Nabanita Mukherjee, Brittany L. Carroll, Jeffrey L. Spees, Eugene R. Delay

The Figure legends in the article were incorrect. The correct legends are available below.

**Figure 1. AMF protects against CYP-induced decreases in taste sensitivity.** This figure depicts sucrose detection thresholds, before and after saline, CYP, AMF or AMF/CYP injection. The graph shows the mean (± SEM) threshold concentration of sucrose (Y-axis, log scale) across days post-injection (X-axis). Days −1, −2 indicate thresholds before the injection. CYP-injected mice showed significant elevations of sucrose detection thresholds on days 2-5 and 9–15 after injection, while AMF/CYP-injected mice showed significant elevation in sucrose detection thresholds on days 9-11 post-injection (*** P<0.001;** P<0.01;* P<0.05).

**Figure 2. AMF protects fungiform papillae with and without pore.** (A) Mean (± SEM) number of all fungiform papillae across days post-injection. CYP injection significantly decreased the total number of fungiform taste papillae on post-injection days 4, 7 and 10 compared with saline control mice. AMF/CYP-injected mice had significantly more papillae compared to CYP-injected mice on days 4 and 7. To the right of the graph are examples of a fungiform papilla without (upper image) and with a pore (lower image). The red arrow identified the opening of a pore. (B) Mean (± SEM) number of fungiform papillae with a taste pore across days post-injection. There was a significant drop in the number of fungiform taste papillae with pores on days 4, 7 and 10 compared with saline controls. AMF/CYP groups had significantly more papillae with pores on days 4, 7 and 10 compared to CYP groups (*** P<0.001;** P<0.01;* P<0.05).

**Figure 3. AMF improves overall morphological index of fungiform taste buds.** (A) Representative bright field images of H and E stained fungiform papillae and taste buds at Days 4, 7, 10 and 16 post-injection of saline, CYP or AMF/CYP-injected mice. Morphologically intact taste buds with organized mass of taste cells (indicated by black arrow) are abundant in saline-injected (control) mice. There was a disruption in the organization of taste buds, seen as an absence of organized mass of taste cells (indicated by green arrow) in CYP-injected mice on day 4 which do not recover until day 16 post-injection. AMF/CYP-injected mice do not show this disruption except on day 7. Scale bar=25µm. (B) Morphological index of fungiform papillae across days in saline-, CYP- or AMF/CYP-injected mice. The morphological index is expressed as p/P, where p=proper taste buds and P=Total number of taste buds. There was a significant decrease in the morphological index for CYP-injected mice on days 4, 7 and 10 compared to saline controls. AMF/CYP mice revealed a protective effect of AMF on days 4 and 7 (*** P<0.001;** P<0.01;* P<0.05).

**Figure 4. AMF protects circumvallate taste buds from the effects of CYP.** (A) This image shows a representative trench of a normal circumvallate papilla with taste buds. The red-boxed area shows the region which is magnified to observe detailed morphology of taste buds within the circumvallate papilla. (B) Images of circumvallate taste buds in saline-, CYP- and AMI/CYP-injected mice. There was no morphological change in the circumvallate taste buds of saline mice across days. However there were open-spaces (red arrow) inside the taste buds on days 7 and 10 in CYP-injected mice. AMF/CYP–injected mice showed similar “open spaces” (red arrow) only on day 7. Scale bar=25µm.

**Figure 5. Mean (± SEM) number of BrdU-positive cells in the basal layer of fungiform papillae and taste buds, one wall of each circumvallate trench, and a sample area of the lingual epithelium in Saline (Day 0), and CYP and AMF/CYP mice on days 4, 7, 10 and 16 post–injection.** (A) BrdU-positive cells in the basal layer of fungiform taste papillae and taste buds of each drug condition. (B) BrdU-positive cells in the basal layer of one wall of a circumvallate trench for each drug condition. (C) BrdU-positive cells in non-taste lingual epithelium of each drug condition (*** P<0.001;** P<0.01;* P<0.05).

**Figure 6. Representative images and summary graphs of Ki67-positive cells (red) in fungiform taste papillae with taste buds and in taste buds in circumvallate trenches of saline-injected mice (Day 0) and CYP- or AMF/CYP-injected mice on days 4, 7, 10 and 16 post-injection.** There was a reduction in the number of Ki67-positive cells in the basal layer of fungiform taste papillae and the basal layer of circumvallate taste buds on day 4 in CYP-injected mice but not in AMF/CYP-injected mice. (A) Ki67-positive cells in the basal layer of fungiform taste papilla and taste bud. Sytox green was used as a nuclear marker. Scale bar=25µm. (B) This graph depicts the mean (± SEM) number of Ki67-positive cells counted in the basal layer and fungiform papillae of each group over days in the basal layer. (C) These images show Ki67-positive cells in the basal layer of circumvallate trenches for each drug condition at each time point. Scale bar=50µm. (D) This graph depicts the mean (± SEM) number of Ki67-positive cells counted in the basal layer of one wall of each circumvallate trench profile over days in each drug condition (*** P<0.001;** P<0.01;* P<0.05).

**Figure 7. Representative images of PLCβ2-positive cells (red) in fungiform and circumvallate taste buds of saline-injected mice (Day 0) and CYP- or AMF/CYP-injected mice on days 4, 7, 10 and 16 post-injection.** (A) In fungiform taste buds, there was a reduction in the number of PLCβ2-positive cells on days 4, 7 and 10 in CYP-injected mice and on day 7 in AMF/CYP-injected mice. Sytox green was used as a nuclear marker. Scale bar=25µm. (B) This bar graph illustrates the mean (± SEM) number of PLCβ2-positive cells in fungiform taste buds across days in each drug condition. (C) In circumvallate taste buds, there was a reduction in the number of PLCβ2-positive cells on days 7 and 10 in CYP-injected mice and on day 7 in AMF/CYP-injected mice. Scale bar=50µm. (D) The bar graph illustrates the mean (± SEM) number of PLCβ2-positive cells in circumvallate taste buds of one wall of each trench profile examined across days in each drug condition (*** P<0.001;** P<0.01;* P<0.05).

