# Supplementary material for: Pre-Treatment with Amifostine Protects against Cyclophosphamide-Induced Disruption of Taste in Mice
Source: PLoS One. 2013 Apr 23;8(4):e61607. doi: 10.1371/journal.pone.0061607 (PMC3634019; doi:10.1371/journal.pone.0061607)
Supplement: Figure S1 — Representative images of BrdU-labeled-cells in different areas of tongue on days 4, 7, 10 and 16 post-injection in saline-, CYP- or AMF/CYP- injected mice. BrdU-positive cells are stained in black (indicated by red arrow) and the sections are counterstained lightly with hematoxylin. Any brown staining outside the trench is non-specific staining. There was a reduction in the number of BrdU-positive cells in fungiform taste buds, circumvallate taste papillae and lingual epithelium on day 4 in CYP-injected mice but not in AMF/CYP-injected mice. (a) BrdU-positive cells in fungiform taste buds (indicated by red arrow). Scale bar = 25 µm. (b) BrdU-positive cells in circumvallate taste papillae (indicated by red arrow). Scale bar = 50 µm. (c) BrdU-positive cells in lingual epithelium (indicated by red arrow). Scale bar = 50 µm. (PDF) [file pone.0061607.s001.pdf]

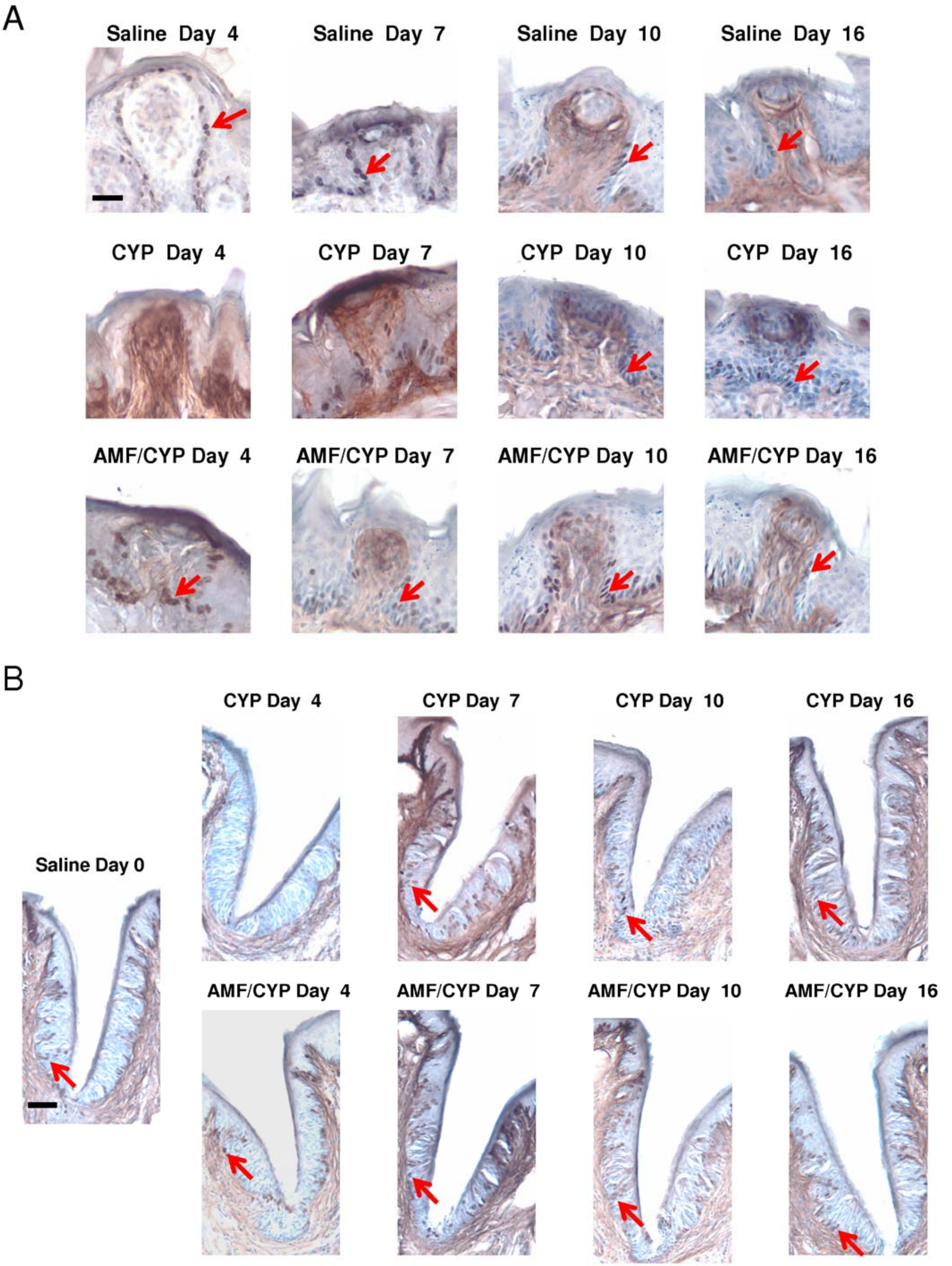

Supplemental Figure 1A and B

C

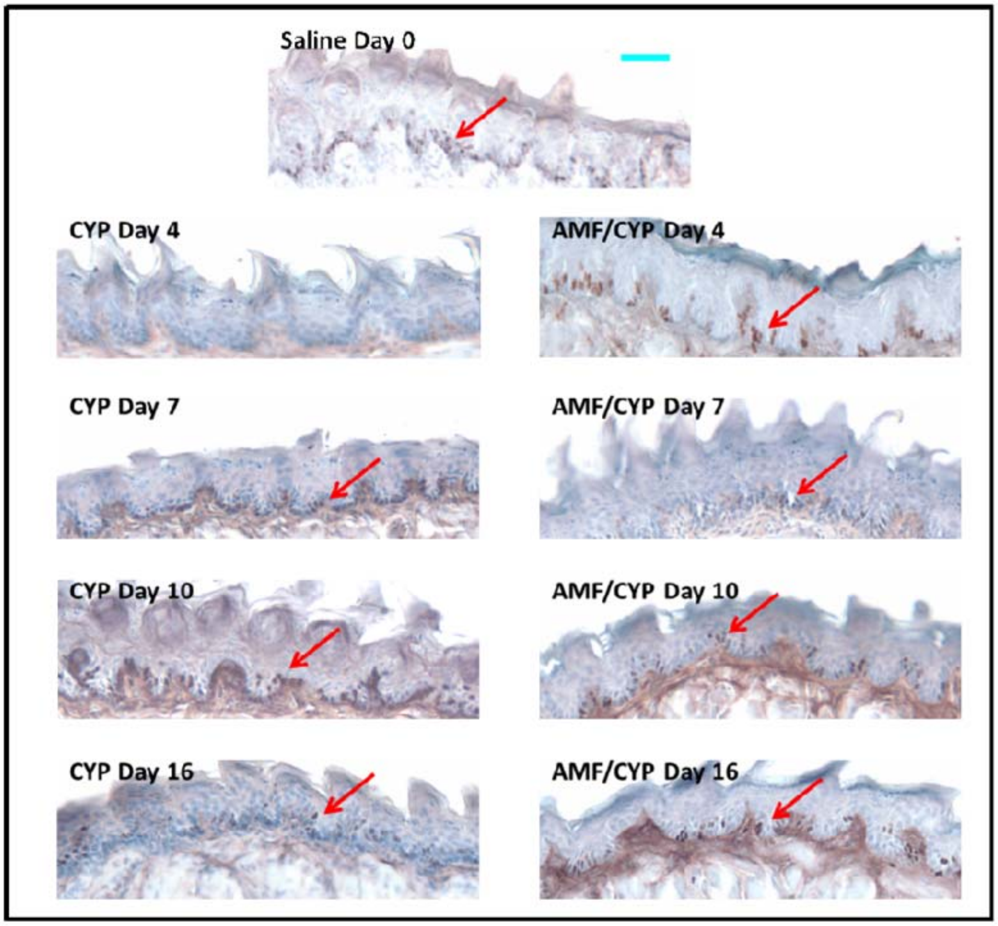

Supplementary Figure S1: Representative images of BrdU-labeled-cells in different areas of tongue on days 4, 7, 10 and 16 post-injection in saline, CYP or AMF/CYP-injected mice. BrdU-positive cells are stained in black (indicated by red arrow) and the sections are counterstained lightly with hematoxylin. Any brown staining outside the trench is non-specific staining. There was a reduction in the number of BrdU-positive cells in fungiform taste buds, circumvallate taste papillae and lingual epithelium on day 4 in CYP-injected mice but not in AMF/CYP-injected mice. (a) BrdU-positive cells in fungiform taste buds (indicated by red arrow). Scale bar=25μm. (b) BrdU-positive cells in circumvallate taste papillae (indicated by red arrow). Scale bar=50μm. (c) BrdU-positive cells in lingual epithelium (indicated by red arrow). Scale bar=50μm.

Supplemental Figure 1C
